# SOA-Based Model for Value-Added ITS Services Delivery

**DOI:** 10.1155/2014/983109

**Published:** 2014-06-10

**Authors:** Luis Felipe Herrera-Quintero, Francisco Maciá-Pérez, Diego Marcos-Jorquera, Virgilio Gilart-Iglesias

**Affiliations:** ^1^Faculty of Engineering, Catholic University of Colombia, Avenida Caracas No. 46-72, Bogotá, Colombia; ^2^Computer Science Department, University of Alicante, Carretera San Vicente del Raspeig s/n, 03690 Alicante, Spain

## Abstract

Integration is currently a key factor in intelligent transportation systems (ITS), especially because of the ever increasing service demands originating from the ITS industry and ITS users. The current ITS landscape is made up of multiple technologies that are tightly coupled, and its interoperability is extremely low, which limits ITS services generation. Given this fact, novel information technologies (IT) based on the service-oriented architecture (SOA) paradigm have begun to introduce new ways to address this problem. The SOA paradigm allows the construction of loosely coupled distributed systems that can help to integrate the heterogeneous systems that are part of ITS. In this paper, we focus on developing an SOA-based model for integrating information technologies (IT) into ITS to achieve ITS service delivery. To develop our model, the ITS technologies and services involved were identified, catalogued, and decoupled. In doing so, we applied our SOA-based model to integrate all of the ITS technologies and services, ranging from the lowest-level technical components, such as roadside unit as a service (RSU_AA_S), to the most abstract ITS services that will be offered to ITS users (value-added services). To validate our model, a functionality case study that included all of the components of our model was designed.

## 1. Introduction

The steady increases in the population and advances in transportation systems have become the key factors in the growth of transportation. The population growth has especially been felt in increased traffic congestion, which has reduced both the efficiency of the transportation infrastructure and its management. In light of this situation, problems such as mobility, fuel consumption, contamination, travel time, and unsafe roads have increased [1, 2].

For these reasons, intelligent transportation systems (ITS) have been designed to address these kinds of problems and to apply information communications technologies (ICT) to the integral management of transportation systems [3]. ITS provide support for improving transportation system operation services, such as traffic management, commercial vehicle operations, transit management, and information to travelers, among others [4].

However, the ever increasing numbers of ITS technologies and their systems and heterogeneity mean that current ITS proposals are characterized by tightly coupled, ad hoc solutions. Therefore, their services are specific, difficult to integrate and extend, and difficult for other similar ITS proposals to reuse. This situation prevents the agile management of the transportation infrastructure and decreases the incorporation of new technologies, the changing of existing services, and the generation of new value-added services.

In other domains, such as e-business, manufacturing, and the automotive industry, the service-oriented architecture (SOA) paradigm has been proposed as the most appropriate solution for solving problems in dynamic and heterogeneous environments [5, 6]. In fact, the SOA paradigm, along with web services technology, allows the achievement of key concepts that are highly valuable for ITS, such as integration, interoperability, reusability, and scalability.

In this paper, an SOA-based model for ITS value-added services delivery is proposed. This model follows the key concepts mentioned above and applies them to ITS technologies and their services. To carry out our approach, three phases associated with the identification, cataloguing, and decoupling of ITS technologies and their services are defined and discussed in the following sections. As a result of these phases, a functional vision of the elements that make up ITS is obtained. This vision allows seeing the ITS elements as services ranging from the lowest-level technical components, such as roadside units (roadside unit as a service or RSU_AA_S), to the highest-level ITS components, such as navigation devices. These latter elements will consume the value-added ITS services offered by the value-added ITS services generation center.

To validate our model, a case study associated with a significant ITS service (a parking management system) was designed and implemented to illustrate its application and functionality and includes all of the components of our model.

The remainder of this paper is organized as follows.  Section 2 describes the related work.  Section 3 focuses on the integration methodology for the value-added ITS services delivery.  Section 4 focuses on the SOA-based model proposal.  Section 5 discusses the system architecture.  Section 6 describes the test scenario, experiments, and evaluation.  Section 7 concludes by describing the improvements this system offers over traditional systems and the potential for future work.

## 2. Related Work

In the field of ITS, traditional software methodologies, such as common object request broker architecture (CORBA), remote method invocation (RMI), and distributed component object model (DCOM), have been used to integrate ITS solutions (center to center communications), but they are highly dependent on the programming language, which has provoked the creation of ad hoc systems as well [7]. Likewise, the traditional way to manage many ITS devices is by means of the simple network management protocol (SNMP) (center to device communications), which has begun to change due to the demand for ITS services [7].

According to future requirements, a new generationof standards that go beyond SNMP and CORBA and embrace new technologies and standards, such as SOA and the standard for ITS on European roads (DATEX II), has begun to change the way that ITS services are deployed [8].

From a technical perspective, an SOA is a collection of self-contained services (system functions) that communicate with each other over specified interfaces [9]. From a business perspective, an SOA is a style of multitier computing that helps organizations share logic and data among multiple applications and usage modes.

In light of this, the International Organization for Standardization (ISO) Technical Committee (TC 204) has recently worked on an SOA for the realization of interoperable ITS [10], and a large amount of research demonstrating its importance has been performed. Research such as [11–13] has focused on improving the ITS architecture management by using the SOA paradigm to resolve the interoperability problems, messages, and entities modeled among ITS components.

In addition, SOA has been used to integrate the information from traffic centers. For example, the US national ITS architecture uses this paradigm through the 9010 and 2306 National Transportation Communications for ITS Protocol (NTCIP) protocols [14]. Another example of this is the Easyway project promoted in Europe [15].

However, the SOA paradigm is only extended to support the center-to-center communication, and the ITS infrastructure requires that all their elements be able to support the services-oriented environment. For this reason, our proposal takes the principal technological elements of the ITS and extends them through the SOA paradigm. In this sense, we focused on certain elements that play a key role in ITS service generation. First, we focused on the roadside unit (RSU) that is responsible for monitoring the interaction between the road and the traffic center or between the road and the vehicles.

Second, we focused on the traffic information centers, which are the most important facilities from which the ITS infrastructure is managed. Finally, we focused on the navigation systems, which are usually not able to offer dynamic information, despite there being a wide variety of technologies that offer information from ITS infrastructures.

## 3. Integration Methodology

The fundamental aim of our proposal is to determine how information technologies (IT) should be integrated into ITS to achieve appropriate ITS value-added services delivery.

Several different organizations, actors, countries, aims, interests, technologies, and services participate in the ITS scenario. For this reason, a methodology that addresses the principal ITS requirements is proposed. In doing so, a systematic model of integration and delivery services that considers the most important ITS requirements is obtained.

**Figure 1:**
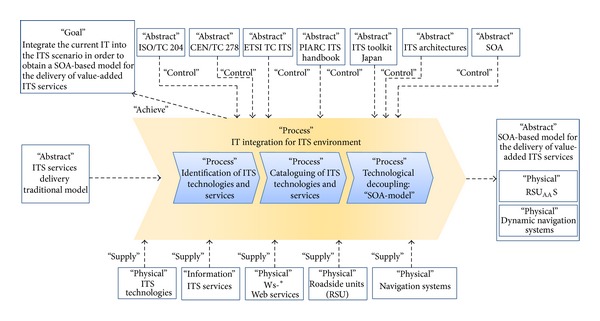
IT integration methodology for the ITS Environment.

The methodology was represented by a unified model language (UML) Erickson and Penker formal notation (Figure 1) and it has a central process that we have called* IT integration for the ITS environment. *This process takes into account the traditional ITS service delivery model and the principal recommendations of the ITS international organizations. Similarly, the process was divided into three internal processes or phases. The first and second phases were termed the* identification of ITS technologies and services *and the* cataloguing of ITS technologies and services*. Both of these phases have been proposed for extracting the principal ITS technological elements that contribute to service delivery from the ITS scenario. The third phase was named* technological decoupling*. It is the basis for our SOA approach, which applies the latest e-business paradigms and N-level architectures to achieve integration, compatibility, interoperability, and scalability in ITS.

The proposed phases were not randomly chosen but rather took into account the principal recommendations of the ITS entities, which seek harmonious ITS applications, the appropriate use of network communication protocols, the application of scalar ITS architectures, and suitable management policies, among other things [16]. We now describe the proposed phases.

### 3.1. Identification and Cataloguing of ITS Technologies and Services (First and Second Phases)

To develop these two phases, the principal ITS proposals associated with ITS standardization (technical committee ISO/TC 204, CEN/TC 278, ETSI TC ITS) were analyzed. The approaches of the principal ITS industrial representatives (ITS America, ITS Japan, and ITS Europe) were also examined to identify the principal technologies and services that make up ITS.

With respect to ITS services, the KAREN project [17] was analyzed because of its insights into ITS user needs. We also analyzed the ETSI TR 102638 proposal [18], which focuses on service delivery to the vehicular network environment. In addition, we included the ISO 14813 [19] proposal by the TC204, which focuses on ITS services. Therefore, our model considered a wide variety of ITS services and new ITS communication environments, such as vehicle to infrastructure (V2I), infrastructure to vehicle (I2V), vehicle to vehicle (V2V), and infrastructure to infrastructure (I2I).

The analysis of the above-mentioned proposals and ITS standards was used to create an exhaustive ITS catalogue, a small part of which is shown in Table 1. The catalogue contains a brief summary of the affinity among the most important ITS services and the ITS technological elements that support them. It is worth noting that the ITS services shown are focused on three principal ITS factors: safety, efficiency, and comfort. The catalogue includes the scope of the service, which can be primary or secondary. The primary scope is associated with the principal ITS communication environments in which the ITS services will be deployed. The secondary scope is associated with the rest of the ITS communication environments and is different from the primary scope.

The ITS technologies in the catalogue have been classified into five categories:* fundamental *(■), without which the ITS services cannot be deployed;* used *(●), which use mature technologies for service delivery;* support *(▼), which are indirectly used to maintain service delivery;* alternative *(▸), whichdecrease communication channels deficiencies; and new technologies (**♦**), which are oriented towards cooperative vehicular systems.

Because it is used to support many ITS services, the roadside unit (RSU) is one of the ITS technological elements from Table 1 that can be highlighted. Indeed, in [20–22], the RSU is emphasized as an important device that contributes to sustainable improvements in the operational efficiency of traffic management.

Now that the ITS technologies have been identified and catalogued, the third phase will be described.

### 3.2. Technological Decoupling Phase

In this phase, the decoupling proposal for ITS technologies, which is the basis of our SOA model's approach to integrating ITS and service delivery, will be presented. This phase takes into account the ITS decoupling proposals presented in [3, 23], from which it was possible to identify the principal ITS technological subsets that presented similar functionalities for supporting ITS services. For example, the ITS technologies that focus on control and monitoring, information processing, and communications (either outside the infrastructure, inside the infrastructure, or to the ITS users) were classified according to [3, 23].

Our proposal took advantage of these approaches and included the services generated from the ITS technologies involved. Two layers were created by considering such service generation: the service layer and the communication layer. The first layer is focused on service generation, and the second layer supports the first layer by means of communication systems. Both of the layers are made up of technological levels that include the present ITS technologies in the ITS scenario.

The service layer consists of three levels: the* monitoring level, the business level, *and* the user level. *All of these levels classify the ITS technological elements by their functionalities. The communication layer contains only one level, called the transverse communication level.

The layers and their levels are described below to establish the starting point of our SOA model proposal.

#### 3.2.1. Service Layer

*(a) Monitoring Level*. This level includes the ITS technologies that are associated with monitoring or watching the ITS infrastructure. For this reason, it includes systems based on* camera networks* (CCTV),* wireless sensor networks* (WSN),* vehicular ad hoc networks* (VANETs, which function as mobile sensor networks),* radiofrequency identification* (RFID), sensors that use* dedicated short-range communications* (DSRC), and infrared sensors that use* continuous* air-interface, of long and medium range (CALM IR). As was seen in Table 1, the RSU plays a key role in integrating the data belonging to several different ITS services that use some of the monitoring systems. Therefore, these systems have some kind of interface, such as CCTV-RSU, WSN-RSU, DSRC-RSU, and RFID-RSU, and the infrastructure data can be gathered by the traffic information center through these interfaces.

*(b) Business Level*. This level is made up of the suitable ITS technologies that lead to creation and delivery of ITS services. Hence, the systems that gather, process, and store the information delivered by several different sources (several monitoring systems) were considered.

In the ITS scenario, the traffic information center (TIC) also plays a key role because a wide variety of the transportation-associated parameters can be controlled, managed, and monitored from it. For example, the vehicular flow produced on the highways can be controlled from a TIC, and it is possible to keep in touch with several critical centers, such as emergency centers, hospitals, and police departments. According to these arguments, the business level is made up of a large number of technological elements, such as database systems, storage systems, application servers (grid systems), and legacy systems. All of these components generate a large quantity of information that is used for providing several different ITS services. 

*(c) User Level*. This level includes the ITS technologies through which the ITS users will be able to consume ITS services. In this way, several different technologies that fulfill user requirements have been divided into the final user systems, the emerging corporate systems, and the systems that deploy visual information directly in the infrastructure.

The first category includes systems such as personal digital assistant (PDAs), smartphones, laptops, navigators, and notebooks. For the second category, it is important to emphasize that, in the near future, customers (pedestrians, travelers, car drivers, and ITS entities) will embrace retail techniques, such as customer relationship management (CRM) systems and the enterprise resource planning (ERP) [24]. Both of these techniques support and enhance customer and organizational relationships to provide better ITS value-added services.

Finally, variable message sings (VMS) systems are included because they display visual information in the infrastructure directly, giving to ITS user certain information about the road.

The users of this level are the many entities that can act as ITS users, such as hospitals, police, roadside emergency assistance centers, car drivers, pedestrians, and travelers, among other ITS users.

#### 3.2.2. Communication Layer

This layer follows the TC 204 guidelines, specifically those from working group 16 (WG 16). Its aim is primarily to support the services generated by the services layer. 

*(a) Communication Transverse Level*. This level supports the ad hoc functionality of each communication system used to achieve the service delivery. Similarly, several communication technologies (wired, wireless, or internetworking) have been included. In the ITS context, the CALM initiative [25], which is still under development, has been taken into account because it includes a large variety of ITS communication technologies. For example, wired and wireless technologies, such as Zigbee, DSRC-RFID/CALM IR, WAVE, CALM M5, Wi-Fi, Ethernet, and Coaxial, and protocols such as RS232, RS422, and RS485 (which is used by VMS), were included. In the same way, the internet working technologies, such as routers, hubs, and switches, were incorporated because they provided connectivity among the levels.

This level uses either the mature communications networks or the emergent communication network to satisfy the service delivery. For this reason, technologies based on general packet radio services (GPRS) or on high speed packet access (HSPA) were considered. In addition, it is worth noting that, in this level, the new long term evolution (LTE) approach [26] will be incorporated as soon as possible. It will provide a new scheme for the ITS value-added services delivery through features such as high speed, bandwidth, sensitivity, and distance coverage.

Finally, the communication technologies based on positioning, such as the global positioning system (GPS) and the geographical information system (GIS), were included.

**Figure 2:**
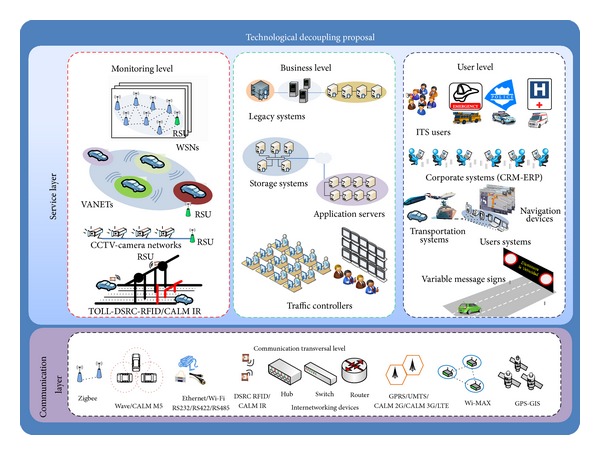
Technological decoupling proposal.

The basis for the SOA model will be the technological elements that have been decoupled in their layers and the levels obtained from the decoupling proposal (Figure 2).

## 4. SOA-Based Model Proposal

By incorporating the SOA paradigm into the technological decoupling proposal, it will be possible to obtain the loosely coupled ITS technologies belonging to each of the technological levels that have been proposed. The principal benefit of our SOA approach to the ITS environment is its ability to deal with various heterogeneous systems and to reduce the technological constraints caused by the ad hoc systems. In doing so, the service delivery will be achieved appropriately.

The SOA paradigm fulfills the standardization requirements demanded by the ITS sector. In a recent publication [10], even the TC 204 has proposed it for exchanging data among several different traffic information centers.

**Figure 3:**
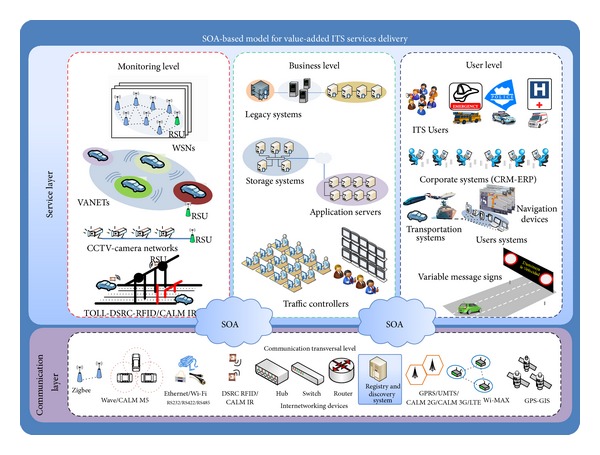
SOA-based model for value-added services ITS delivery.

Specifically, our proposal is focused on extending the SOA paradigm to the global ITS scenario (Figure 3). Therefore, this paradigm will be used not only for the TIC interconnection but also for integrating the significant ITS technological elements under the service scheme.

In this context, certain key concepts, such as integration, interoperability, scalability, and compatibility, can be achieved either by the ITS technological elements or by the other ITS systems that compose the ITS scenario.

As can be seen in Figure 3, three new elements have been added that conceptually represent our loosely coupled proposal, all of which are associated with the SOA paradigm. Two of them are small clouds that integrate the proposal levels, and the third element is the registry and discovery system, which has been enclosed in a picture located in the communication transverse level.

Web services technology was used to apply the SOA paradigm to the ITS context because it is a powerful method for achieving the key concepts mentioned earlier.

Applying the SOA paradigm over the decoupling proposal will generate four interfaces that relate the technological levels. These interfaces support the services environment among the technological levels.

The* first interface* relates both the monitoring level and the business level, which provokes the creation of two kinds of services: the monitoring services and the management services. The monitoring services are focused on gathering the information belonging to the ITS monitoring systems, and the management services are focused on managing the ITS technological elements belonging to the ITS scenario.

The RSU has been taken into account for this interface because of its key role in the monitoring systems. For this reason, we apply the SOA paradigm to this element and obtain a new concept called the RSU_AA_S (the RSU as a service). The RSU_AA_S has new capabilities, such as publishing, understanding, and consuming the web services associated with the monitoring systems.

The* second interface* focuses on the generation of value-added ITS services and uses the business level for keeping the information sent by the RSU_AA_S. It is worth noting that this interface also uses the information provided by the legacy systems for the value-added ITS service generation.

The* third interface* relates both the business level and the user level by means of the SOA paradigm.

In this interface, the final user devices, the corporate systems, and the VMS systems and ITS entities (police, emergency assistance centers, and so on) are highlighted because they will consume the value-added ITS services.

The* fourth interface* is directly associated with the communication transverse level that supports all the levels belonging to the service layer. Its principal element, based on the SOA environment, is the registry and discovery system of services.

The interfaces that are part of our SOA-based model follow the Matchmaker pattern [27], which means that the broker functions are carried out by the registry and discovery system.

As of these interfaces mentioned above, the processes associated with the SOA paradigm,* publication*,* discovery,* and* consumption,* will now be described.

### 4.1. Publication Process

The fundamental aim of this process is service publication (specifically, the monitoring services and management services generated by the monitoring level and value-added ITS services generated by the business level). In this way, this process publishes the monitoring services, the management services, and the value-added ITS services by means of the registry and discovery systems, which can be implemented using the universal description, discovery, and integration (UDDI) approach [28]. As regards the monitoring level, it should be noted that the service providers for the business level are the RSU_AA_Ss. As regards the business level, the service providers are the application servers. To carry out such publication processes for both levels, the web services definition language (WSDL) sheet exchanges and the use of simple object access protocol (SOAP) messages are needed. For this reason, the system belonging to the monitoring level described previously incorporates a system that can publish, discover, and consume web services. In doing so, systems such as the wireless sensor networks (WSN) incorporate an RSU_AA_S (WSN-RSU_AA_S), the VANET systems incorporate an RSU_AA_S (VANET-RSU_AA_S), the CCTV systems incorporate an RSU_AA_S (CCTV-RSU_AA_S), the systems based on a DSRC or RFID incorporate an RSU_AA_S (DSRC-RFID-RSU_AA_S), and on so on.

In the case of the application servers, the SOA paradigm has also been considered. For this reason, their services (the ITS value-added services) can be published, discovered, and consumed.

### 4.2. Discovery Process

The principal aim of this process is to discover the published services on the broker (UDDI). This process is carried out when the publication process has finished.

To discover which services have been published, our model uses the* lookup services* of the storage systems (the lookup-monitoring services), the application servers (the lookup-management services), or the technological systems belonging to the user level (the lookup-value-added ITS services). In this way, the discovery process is achieved when these services are located.

It should be noted that the management services play a key role in the context of the model because the calibration and the management of the monitoring systems will be performed in a services environment. This consideration means that the RSU_AA_Ss will be managed from the business level.

In addition, the RSU is typically managed by means of SNMP protocol at present, which limits the transference of large quantities of data. For this reason, a web services solution was embraced for implementing the key concepts previously mentioned.

### 4.3. Consumption Process

The fundamental aim of this process is to allow the consumption of the services either by the monitoring services, the management services, or the value-added ITS services. Following our proposal, the business level consumes the services offered by the monitoring level, which means the services offered by the different types of RSU_AA_S. Similarly, the user level consumes the services offered by the business level (the value-added ITS services), which means the services offered by the application servers.

In this way, an ITS user who wants to consume a value-added ITS service must perform a service query associated with the desired ITS service. A response message with the associated information for the requested service will then be obtained. Similarly, the same situation is applied to both the monitoring and management services. When a service query is carried out, a message response will also be obtained, but it will be associated with either a monitoring service or a management service.

**Figure 4:**
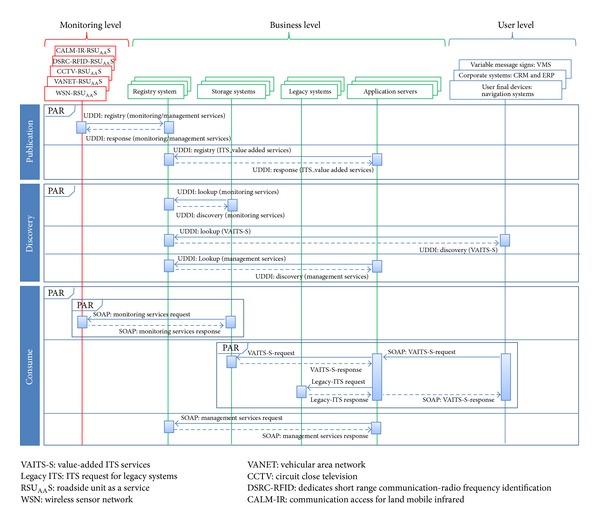
SOA-based model sequence diagram for ITS services delivery.

To describe the process that follows the SOA-based model appropriately, a sequence diagram is depicted in Figure 4.

The layers, levels, interface, and SOA processes of the decoupling proposal are presented in Table 2. Table 2 includes the loosely coupled mechanisms that are used to accomplish the ITS service delivery.

Similarly, Table 2 presents the kinds of services that are used in our SOA-based model, along with the ITS service providers and the ITS service consumers. As can be seen in Table 2, there are some internal services, which are denoted by the ∗ symbol. These services have been taken into account because they supply our model with systems that guarantee service deployment under the SOA scheme.

In addition, an important question about the real-time response of our model is crucial for both the user ITS and the systems that use their generated services. For this reason, an important fact associated with the ITS service delivery should be highlighted. Currently, the ITS service delivery is highly dependent on the ITS technologies installed in the infrastructure, especially when each service can use one technology or a combination of several technologies. In addition, the ITS technologies can have response times that are quite different. This situation can be seen in [29] in which a value-added ITS service can have several different response times. For example, the technologies belonging to the VANET scenario have minimal response times because most of the ITS services are safety oriented. However, the VANET scenario has time delays that range from thirty seconds to five minutes [30]. In the same way, the ITS services that use radio data systems (RDS) are slow (10 minutes) [29].

In keeping with these facts, our model will offer the value-added ITS services within 60 seconds, which means a low latency for the ITS user.

Finally, the services provided by our model can be consumed at the user level specifically by navigation systems. This consideration means that the navigation systems will incorporate dynamic services for the ITS user.

## 5. System Architecture

**Figure 5:**
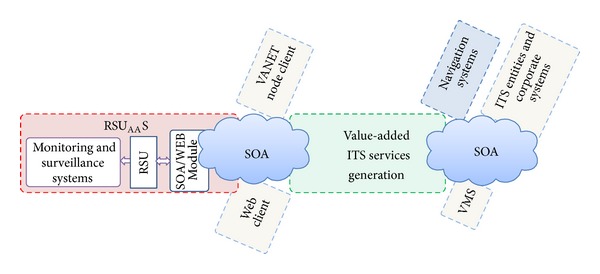
General architecture proposal.

Each of the elements of the ITS scenario is considered by the system general architecture. The principal aim of the architecture is to support the proposed model by offering the functionality elements as services. This paper focuses on three of the main ITS components: RSU_AA_S (the monitoring level), value-added ITS service generation system located in the business level (focusing on the traffic center services), and navigation systems (the user level). In addition, the architecture takes account of the SOA pattern previously mentioned in the model description. The proposed architecture (Figure 5) for each ITS element is based on the conceptual model of the e-business architecture proposed in [31] and includes the ITS architectural components that have been defined by the main ITS organizations, such as [32–34] and the ISO/TC 204.

**Figure 6:**
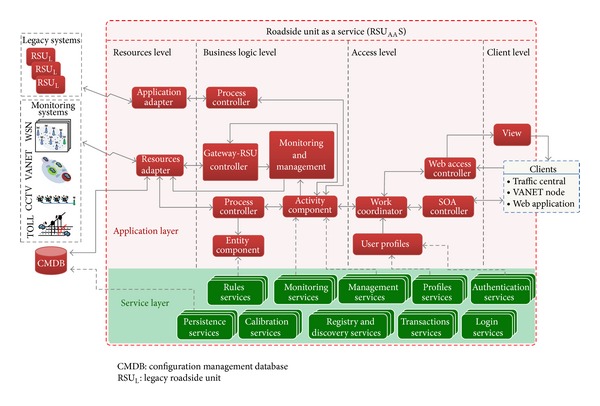
RSU_AA_S architecture.

The RSU_AA_S architecture (Figure 6) is made up of two functional layers: the service layer and the application layer. The service layer supports the application modules of the layer above through a set of standard services (e.g.,* rules, persistence, calibration, monitoring, registry, discovery, and management, among other services*). The application layer has been divided into four distribution levels [28]: resources, business logic, access, and client.

The resource level, independently of technology, provides the necessary information to carry out the monitoring processes. In this sense, the monitoring systems themselves, the legacy RSUs, and the RSU_AA_S databases were taken into account.

The business logic level is made up of several different components that encapsulate the RSU_AA_S functionality. This level obtains, processes, stores, and prepares the data resources that are consumed in the access level.

The access level controls the external ITS users' access by means of services that ensure the protection of the system. In the same way, this level coordinates all of the activities belonging to the RSU_AA_S consumers and exchanges information with both the business and the client levels.

As regards the client level, an interface to exchange the information has been considered. It takes into account the information presentation, input and output data, and application navigation flow. When the clients access the RSU_AA_S by means of the SOA controller, the system directly allows them to get into the access level because the information presentation tasks are not necessary.

**Figure 7:**
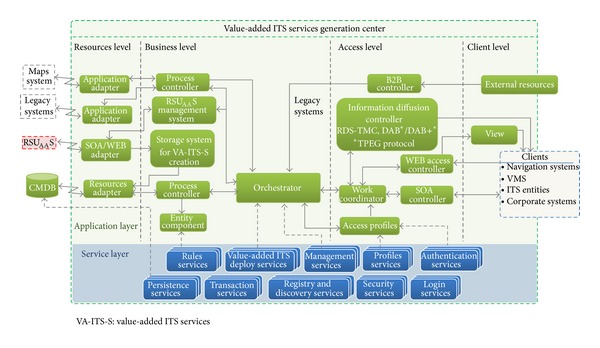
Value-added ITS services generation system architecture.

The value-added ITS service generation system is described in Figure 7. It takes advantage of the RSU_AA_S and other elements to build the value-added ITS services. Specifically, the system is an instantiation of the information traffic center (principally of the ITS services), which was analyzed in the previous sections.

It is worth noting that, from this center, the RSU_AA_S functionality can be maximally exploited, given the fact that these elements are services-oriented.

There are some similarities between the services provided by the RSU_AA_S and those provided by the value-added ITS service generation system, as shown in the service layer (Figure 7). However, some of the middleware services for supporting the new application modules of the layer above are focused on the traffic information services.

As can be seen from Figure 7, the architecture also includes some levels relating to the e-business architecture: the resource level, the business level, the access level, and the client level.

The resource level includes a new application module that, by means of the SOA paradigm, integrates the functionality of services provide by the RSU_AA_S. The business level includes new application modules that allow the adequate management of the RSU_AA_S, information storage, and service generation (services orchestration). The access level includes an information diffusion controller to distribute traffic information via radio channels: RDS-TMC, digital audio broadcasting (DAB), and DAB+. Finally, the client level provides the necessary interfaces for value-added ITS service consumption by end users.

It should be noted that the client level has an external resource module that is important because it allows integration with other traffic information services, in which the 2306 NTCIP protocols or the emergent DATEX II approach could be applied.

The last of the architectural components is a navigation system that can be used by the ITS user to consume the value-added ITS services. Basically, its architecture is based on the component approach [35].

**Figure 8:**
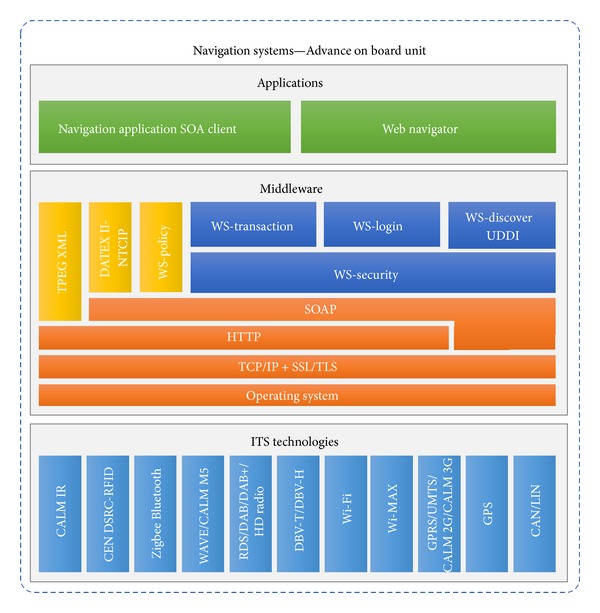
Navigation system architecture.

As can be seen from Figure 8, the conceptual architecture for the navigation system includes the CALM proposal and the middleware approach, which are necessary for service delivery. In addition, a module based on DAB systems for supporting the transport protocol experts group (TPEG) has been included, although it currently is under development, along with the DATEX II module. However, our SOA module was designed to support the services environment that provides the service consumption.

## 6. Test Scenario and Experiments

In this section, a test scenario and a set of experiments designed to validate the proposed model are detailed. The scenario was based on the value-added ITS services that can be produced by a parking management system. This scenario offered us a large variety of ITS technological elements that could be integrated under our approach. We focused on the associated valued-added services that facilitate the user's ITS task of finding a parking place at the end of a trip, along the way, and on the trip. This significant service was selected because it helps the ITS users solve problems associated with mobility and fuel savings and decrease travel time [36]. In addition, it is suitable for evaluating our SOA-based model.

The parking areas belonging to the campus of the University of Alicante (UA) were used as a test environment. According to the proposed architecture and the associated layers of the model, the ITS scenario for some of the experiments will be described.

### 6.1. Service Layer

#### 6.1.1. Monitoring Level

Emerging monitoring technologies based on wireless sensor networks (WSN) were used at this level because of their characteristics: ubiquity, low power consumption, small size, processing speed, and ability to cover large areas. In addition, WSN are ideal for supporting the proposed service. In this way, twelve nodes that constituted the wireless sensor network were used for our experiment. A* crossbow solution,* called MicaZ [37] in the field of WSN, was used for monitoring the parking lot. This solution incorporated several sensors; we used a photoresistor to detect the status of the parking places.

Software that ran on each sensor node and was capable of detecting the status of the parking places was developed for our prototype. The* TinyOS* operating system and* nesC* programming language were used to implement it. The software operated on demand, which means that each node transmitted information on when the parking place was occupied or free and information for maintaining the network topology (sporadic messages). The XMESH protocol [31] was used to make the routing.

**Figure 9:**
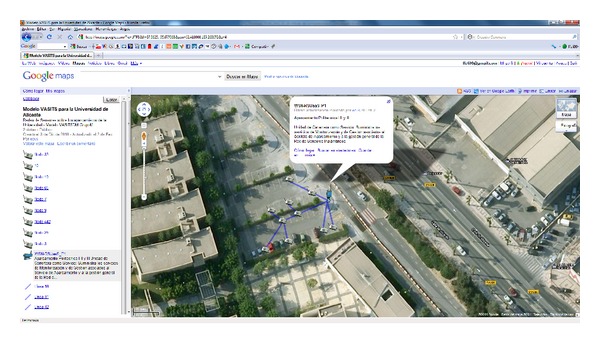
University of Alicante-Parking for the Polytechnic School.

Our first experiment was designed to evaluate the software and routing protocol we developed. Therefore, we analyzed its metric using the exponentially weighted moving average (EWMA) estimation algorithm [38]. This metric minimizes the total cost to transmit a packet from a node located in the parking place to the base station. The selected scenario to test this software was the parking for the Polytechnic School, whose capacity was 98 places (Figure 9). The WSN was deployed there.

In this scenario, it is worth noting that, at the moment of carrying out the deployment process for the WSN, most of the retransmissions generated among the nodes were necessary for determining the neighbors belonging to the WSN. In this way, different routing tables and an estimated pattern for link qualities among the nodes were constructed. This pattern was a key factor for finding the route that minimized the total number of transferences, which is reducing power consumption for each node.

The scenario had twelve nodes: ten to determine the status of parking places, one for gathering the traffic associated with the sensor network (the sniffer), and one for the base station, which was connected to the RSU.

**Figure 10:**
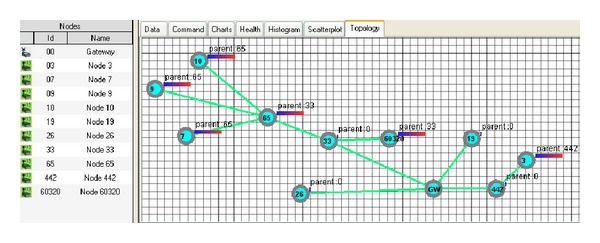
WSN Topology for Politecnica's School Parking.

To determine the WSN topology (Figure 10), the* Moteview* tool was used. The topology used the minimum transmission metric to establish the organization and provide the information associated with the parking places.

**Figure 11:**
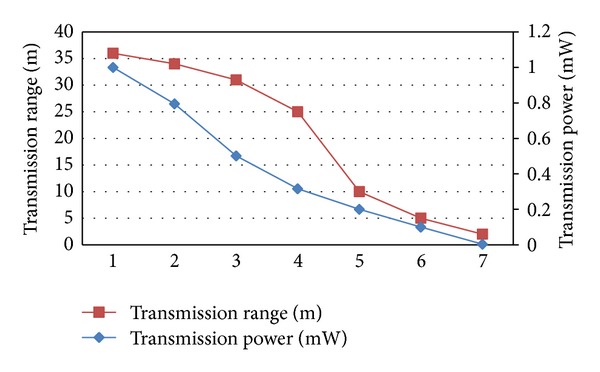
Relationship between the transmission range and the node power.

By evaluating the WSN, the dependence between transmission reliability and the power used by each node could be observed. Therefore, several different powers were set up, and the transmission ranges were observed (Figure 11). A power of 0.1 mW was selected because it was the most appropriate for covering the distance between each parking space (2.5 meters).

Finally, data packets containing the status of the parking space were obtained, which showed the effectiveness of the minimum transmission algorithm (Table 3).

From Table 3, it can be concluded that the nodes most remote from WSN-RSU (7, 9 and 10) had more difficulty transmitting the information associated with the parking space, while those directly connected to the base station (19, 26, and 33) had a higher probability of success in transmitting information. Finally, data packets regarding the status of the parking space were obtained, showing the effectiveness of the algorithm of minimum transmission (Table 3).

From Table 3 it can be concluded that the most remote from nodes WSN-RSU (7, 9, and 10) had more difficulty transmitting the information associated with the parking space, while those directly connected to the base station (19, 26, and 33) had a higher probability of success in transmitting information. However, node 442 (which was connected directly to the WSN-RSU) had low transmission quality, which influenced the cost of the proposed metric.

Having evaluated the performance of the WSN as a parking monitoring system, an RSU_AA_S prototype will now be described and evaluated.

To select the WSN-RSU_AA_S devices, various requirements were taken into account. The devices needed to facilitate deployment along the ITS infrastructure, being as small as possible, incorporate the management protocols of the current RSU (such as SNMP), integrate the standard protocols for communication with the central traffic (or otherwise have the capabilities necessary to implement them), and have network connectivity (either wired or wireless) that allowed remote access.

As mentioned above,* MOXA W321* [33], an embedded device widely used in ITS solutions, was chosen. The device was selected for its hardware and software characteristics, including its ARM microprocessor at 192 MHz and 32 bits, 32 MB of RAM memory, SD card support, low cost, ethernet-serial gateway, Wi-Fi support, uClinux 2.6, C and C++ support, integrated web server (Apache), data encryption features, and two RS232/RS485 serial ports.

To carry out the WSN-RSU_AA_S, multiple applications and services were developed and integrated according to the previously proposed architecture.

At the resources level, an application that managed the data exchange through the serial port between the base node of the WSN and the RSU was developed.

For the business logic level, three applications were developed.

The first application was responsible for controlling the base node and its associated nodes.

The second application supported the generation of monitoring services and stored the data collected by the sensor nodes. This application was quite complex, and its development required the integration of an embedded database (DB) into the device. The DB was used to store data from the WSN, including information related to the status of parking spaces and other items for WSN management. The database selected was SQLite [34]. SQLite provided a compact library with low memory requirements, which was suitable for the embedded devices.

The third application was associated with the activity controller and process controller and provided the information stored in the database when a request was performed.

For the access level, SOA paradigm (WSN-RSU_AA_S) was used to achieve the RSU_AA_S concept. The gSOAP library [35] was used to achieve this, which allowed the implementation of web services in C/C++.

Finally, it should be stressed that the RSU_AA_S published its services in a UDDI registry server, so that customers could find and consume the services offered.

To evaluate the RSU_AA_S performance, its main customer, the value-added ITS services generation center, is first described.

This section is described in greater detail in [39].

#### 6.1.2. Business Level

To implement this level, two applications of our architecture were designed. The first measured the potential of the services generated from the RSU_AA_S and incorporated a transaction service to verify the proper operation of the services offered by the RSU_AA_S. The second application was more general and complex and implemented the functionality of the value-added ITS services generation center.

**Figure 12:**
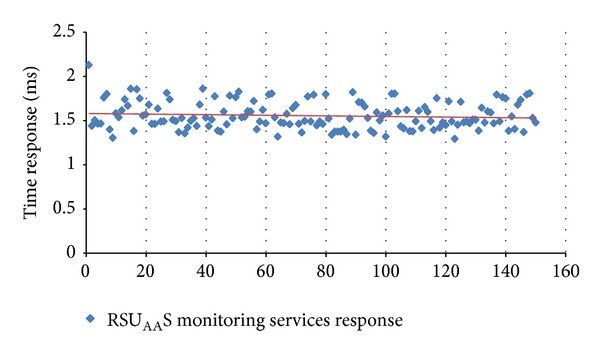
RSU_AA_S Monitoring services response.

For the first application, an experiment to evaluate the RSU_AA_S service response was designed. The experiment consisted of consuming continuously monitored services provided by the RSU_AA_S. In the tests, 150 requests with different time lapses were sent to the RSU_AA_S to analyze the response performance. The device took, on average, 1.56 seconds to process the SOAP messages (Figure 12).

**Figure 13:**
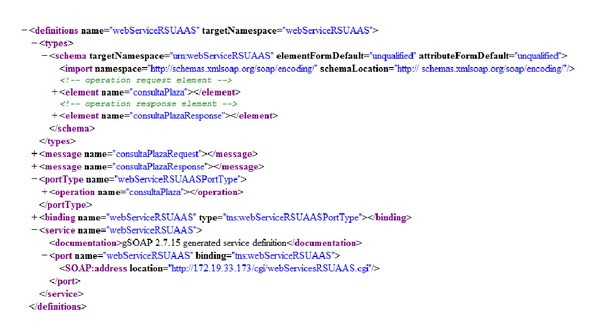
Soap message for the* parkingPlaceQuery* () service.

The SOAP message to the* parkingPlaceQuery* service can be seen in Figure 13.

The* parkingPlaceQuery* service was just one of the twenty-five services that integrated our monitoring services platform. The remaining services were related to setup, including parking places, sensors associated with a parking place, service users and their preferences, reservation of a place, new sensor startup, and others.

**Figure 14:**
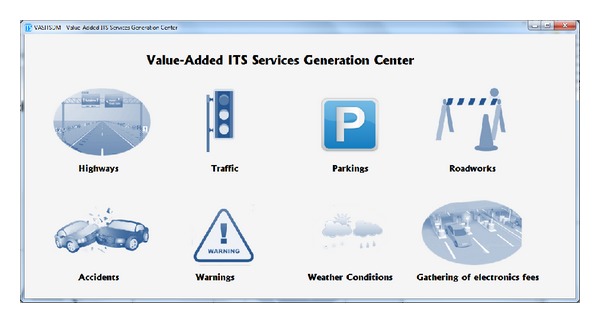
Value-Added ITS Services Generation Center.

The Framework 3.5 NET and C# language were used to generate classes that supported the second application (Figure 14). Several ITS areas were included in this application, although only the implementation of car parking was used for the prototype.

An SOA/WEB adapter for communication with the RSU_AA_S was incorporated into the resource level, as were application adapters that allowed connection to the storage system.

Two servers were used as the computational infrastructure. The first server had two purposes: to support the main application of the traffic information center and to support the storage system monitoring services such as the RSU_AA_S management system. The UDDI registry server and applications that offered the value-added ITS services were supported by the second server.

**Figure 15:**
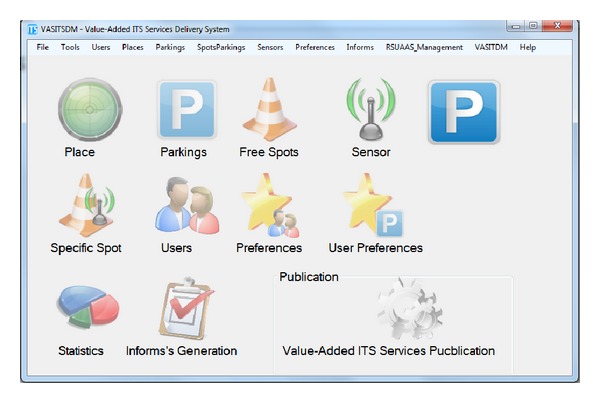
Services Oriented Parking Management Center.

The application environment that acted as the manager of the parking system is shown in Figure 15.

A MySQL database was used by the application to store monitoring systems information.

This application allowed managing parking spaces and places areas, managing the monitoring system itself, and configuring the WSN (the wireless transmission channel, power, number of nodes, node groups, node configuration, and node rebooting).

This method was a new approach to the management of ITS monitoring systems because it used the SOA scheme and not traditional protocols, such as SNMP or simple message transfer protocol (SMTP).

For testing, four WSN management services were evaluated: a node reboot, a power shift, getting the settings from the WSN node, and general data dissemination through the WSN.

**Figure 16:**
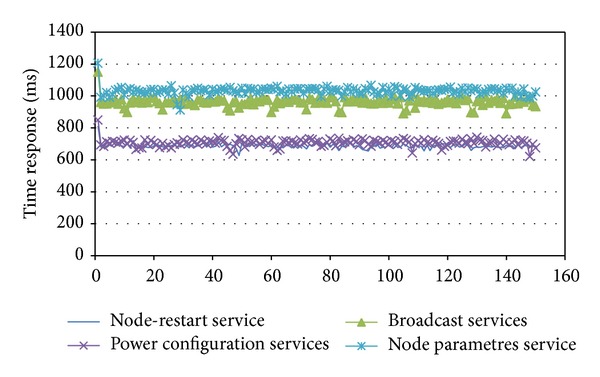
WSN-RSU_AA_S time response.

For each management service in the experiment, 150 requests were sent to the RSU_AA_S. The results (Figure 16) show that the WSN-RSU_AA_S responded efficiently to the requests made from the central traffic information. The power settings service and reboot service responded with average times of 707 and 687 milliseconds. However, the services related to the dissemination of information through the WSN and to getting the sensor node configuration took an average of 963 and 1031 milliseconds, respectively. This delay was due to the response being associated with a great deal of information about the WSN.

**Figure 17:**
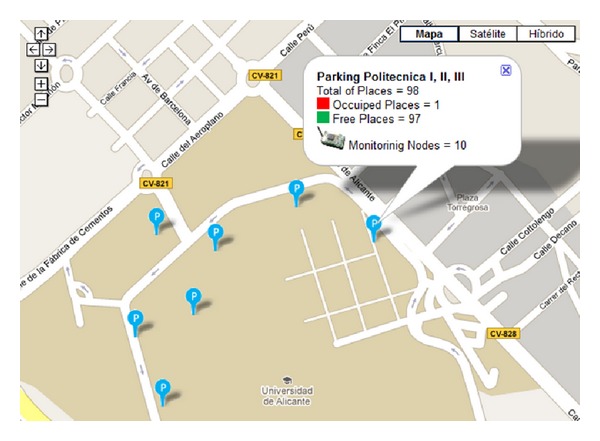
Web application.

Moreover, the integration of generic and open source tools, such as* Google Maps*, was one of the most important features incorporated into the system. This type of solutions allows ITS services that are adequately provided and that can be consumed by a large number of external clients regardless of the browser platform they use (Figure 17).

#### 6.1.3. User Level

Two applications were designed for final consumption of services. The first one was a desktop client application for mobile devices that provided information on free, busy, and not available parking places. The second application offered the same service, but the results were displayed on* Google Maps*.

**Figure 18:**
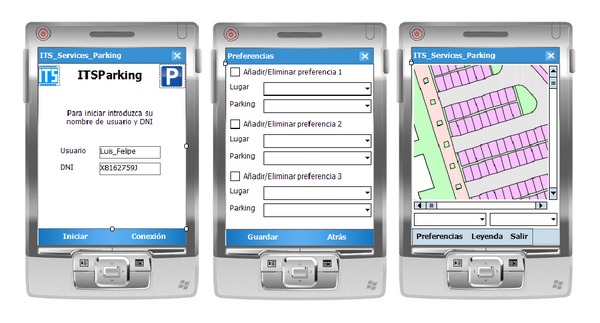
Application's aspect for PDA devices.

The first application (Figure 18) was developed using the.*NET* Compact Framework for mobile devices and C#. This application consisted of a mobile client that queried the value-added ITS services published in the UDDI registry and consumed them to perform its activity.

This application, called* ITSParking*, provided information on parking spaces and allowed users to enter their parking preferences according to their usual customs. This application was available for university users (students, teachers, and administrators) who had registered to use the service.

Both applications were tested on various user devices, such as the* HTC Diamond 2* and* HP iPAQ h6340*.

The experiment concluded that the performance was adequate and fast enough from both the RSU_AA_S and the value-added ITS services management center. The consumption associated with the parking services ranged between 7 and 10 seconds, considering the time it took to offer the RSU_AA_S monitoring services. The margin of 60 seconds initially proposed by our model was achieved.

### 6.2. Communication Layer

For this layer, various communication systems that supported the connectivity of all the elements involved were used. Specifically, we used technologies such as ZigBee, RS232, Ethernet, Wi-Fi, and GPRS/HSPA.

For the SOA scenario, a server UDDI service discovery was used. In this case, the jUDDI-rc4 version was built, and a UDDI server was developed in Java.

**Figure 19:**
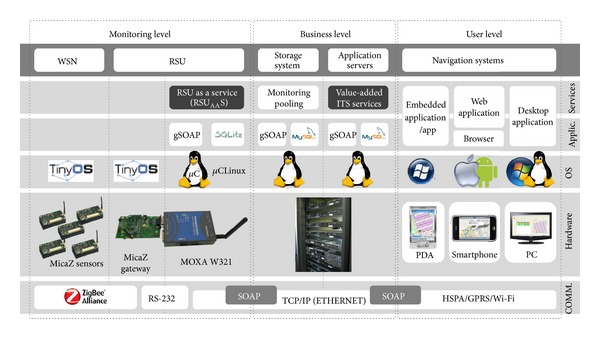
Test scenario for value-added ITS service.

Figure 19 summarizes the proposed test scenario, including the systems and technologies used to develop the prototype. The figure arranges the system elements according to their communications, hardware, operating systems, applications, and services.

## 7. Conclusion

In this paper, we presented, developed, and tested a SOA-based model for the value-added ITS services delivery. Our approach achieved a loose coupling among each of the technologies and services present in the ITS scenario. In light of this, new value-added ITS services could be composed and offered to several ITS components where the navigation system was highlighted. As part of the process, we developed a RSU_AA_S, a new concept for the RSU device that permits showing both monitoring services and management services as web services. To validate the proposal, a valued-added service that facilitates the user's ITS task of finding a parking place at the end of a trip, along the way, and on the trip, was developed. To deploy this service, several ITS technological components were used: new emerging technology (WSN), embedded computer systems, navigation devices, databases, and application servers. This SOA approach opens up a wide range of possibilities in the management of ITS service, such as automatic provision of resources and services ITS on demand based on environment information. Nowadays, we are working in this research line using the proposed model, services, and processes orchestration with BPMS and ontologies applied to traffic management.

## Figures and Tables

**Table 1:** ITS services and technologies catalogue.

ITS technologies and services general catalogue				Communication technologies										Employed systems							
PF	ITS value- added service	Scope service		CALM IR	CEN DSRC/RFID	ZIGBEE	WAVE/CALM M5*	RDS/DAB	DVB-T DVB-H	Wi-Fi a/b/g/n	WiMAX, LTE*	GSM/GPRS	UMTS/HSPA	NS (OBU)	US	SS	APP_S	CCTV_S	CS**	LS	RSU
		Primary	Secondary																		
SAFETY	Warning by variable message signs (VMS)	I2V	I2I				♦				♦	■	■			●	●			▼	■
	Roadwork warning		V2V	▸	▼	▼	♦	▸				●	●	■	▸	●	●		▸	▼	■
	Pedestrian monitoring		V2V	▸	▸	●	♦					▼	▼	■	▸	●	●	▼	▸	▼	■
	Fog warning		I2I, V2V, V2I	▼		▼						▼	▼	■	▼	●	●		▸	▼	■
	Snow warning		I2I, V2V, V2I			▼						▼	▼	■	▸	●	●		●	▼	■
	Approaching emergency vehicle	V2V	V2I, I2V, I2I	▸			♦					▼	▼	■	●	●	●			▼	■
	Cooperative forward collision warning		V2I, I2V	▸		▼	♦			▸	♦			■	●		●				■
	Highway/rail collision		V2I, I2V, I2I	▸		▸	♦					●	●	■	▸	●	●		●	▼	■
	Postcrash warning		V2I, I2V, I2I	▸			♦					▼	▼	■	▸	●	●			▼	■
	Special vehicle detection (tractor, crane, fire truck)		V2I, I2V	▸	▼	▼	♦					■	■	■	▸	●	●		▸	▼	■

EFICIENCY	Medium speed	I2V	V2V		▼	▼	♦				♦	▼	▼	■	▸	●	●		●	▼	■
	Speed control		V2V	▸	▸		♦					▼	▼	▼	▸	●	●			▼	■
	Public transport watching		I2I			●	♦					■	■	▼	▸	●	●	▼	●	●	■
	Borders identification		I2I	▸			♦	●				▼	▼	▸	▸	●	●			▼	■
	Intermodal conteiner tracking		I2I			▼						●	●	■	▸	●	●	▼	▸	▼	
	Parking management: discovery of free parking places	I2V	V2V, V2I		▸	▼	♦				♦	■	■	■	▸	●	●	▼	▸	▼	■
	Hazardous freight Coordination	V2V	V2I, I2V, I2I	●		●	♦					●	●	■	▸	●	▼		●	▼	
	Disaster response management	I2I	I2V				♦		■	■	♦	■	■	▼	▸	●	●		▸	▼	▼
	Vehicles as sensors for gathering data	V2I	I2I	▼		▼	♦			▸	♦	▼	▼	■	▸	■	■	▼		▼	■

CONFORT	Dynamic traffic information (weather, close highways, bridge state, speed limit, etc.)	I2V	V2V, V2I	▼		▼	♦	●		▸	♦	■	■	■	▸	●	●	▼	▸	▼	■
	Weather forecast	I2V	V2V			▼	♦	●		▸	♦	▼	▼	▼	▸	●	●		●	▼	▼
	2D/3D POI visualization	I2V	V2V				♦			▸	♦	●	●	■	▸	●	●		▸	▼	
	Toll detection	I2V	V2V	▼	■	▼	♦			▸	♦	▼	▼	■	▸	●	●		▸	▼	■
	Toll fares payment	I2V	V2I, I2I	▼	■		♦					■	■	■	▸	●	●		▸	▼	■

PF: principal factor; systems and technologies: ■→ fundamental, ●→ used, ▼→support, ▸→ alternative, ♦→ new technological scheme, NS→ navigation systems (on board unit-OBU), US→ users systems, SS→ storage systems, APP_S→ application servers, CCTV_S→ circuit close TV systems, CS→ corporate systems, LS→ legacy systems, RSU→ roadside unit, *→technologies under growth, and **→ new ITS proposal.

**Table 2:** Loose coupling by means of an SOA based model for ITS service delivery.

Kinds of ITS services	Integration and decoupling mechanism	ITS service provider	ITS consumer
Monitoring service	SOA (SOAP)	WSN-RSU_AA_S/VANET-RSU_AA_S/CCTV-RSU_AA_S/DSRC-RFID-RSU_AA_S/CALM-IR-RSU_AA_S (ML)	Storage Systems/Application Servers (BL)
Management services for the RSU_AA_S	SOA (SOAP)	XX-RSU_AA_S (ML)	Application Servers (ML)
value-added ITS services	SOA (SOAP)	Application Servers-Legacy Systems (BL)	Navigation Systems, Corporate Systems, VMS (UL)
Registry and discovery services for the monitoring services*	SOA (UDDI) Service catalogue	Registry and Discover Server (CTL)	Monitoring Services (ML)
Registry and discovery services for the management services*	SOA (UDDI) Services Catalogue	Registry and Discover Server (CTL)	Management Services (NM)
Registry and discovery services for the value-added ITS services*	SOA (UDDI) Services Catalogue	Registry and Discover Server (CTL)	Value-Added ITS Services (BL)

*Internal services; ML**→ **monitoring level; BL**→** business level; UL**→** user level; CTL**→** communication transverse level; XX**→ **can be WSN, VANET, and so forth.

**Table 3:** WSN metric for determining when a parking lot is free or busy.

Node	Node_Pkts	Forwarded	Dropped	Retries	Bat	Q_Tx (%)	Q_Rx (%)	P_C
3	91.67	0	8.33	79.71	2.5	93.33	100	12
7	10.59	88.56	0.85	15.69	2.7	86.67	100	13
9	10.7	84.66	4.64	40.63	2.9	100	86.6	12
10	18.8	80.3	1.61	23.68	2.7	100	86.67	13
19	1.86	97.99	0.15	40.63	2.6	100	86.67	4
26	16.64	82.86	0.4	12.6	2.6	100	100	4
33	14.01	85.79	0.2	31.75	2.9	100	100	4
65	21.43	70.39	8.18	81.37	2.8	93.33	100	8
442	80.07	19.33	0	58.18	2.9	43.67	100	9
60320	1.45	98.4	0.15	38.12	2.7	100	100	8

Q→ quality, Bat→ battery (V), Node_Pkts→ node packets, and P_C→ path cost.
